# High and low Fc IgG-receptor expression in human chronic granulocytic leukaemia cells.

**DOI:** 10.1038/bjc.1982.33

**Published:** 1982-02

**Authors:** A. Harłozińska, J. Potomski, B. Lawinska, A. Noworolska, R. Richter

## Abstract

Discontinuous density-gradient centrifugation was used to separate chronic granulocytic leukaemia (CGL) cells in the chronic phase and blast crisis (BC) into fractions containing granulocytes in individual stages of maturation. The occurrence of the Fc IgG (FcR) and complement-component receptors (CR1 and CR2) in each fraction was estimated. It was established that, with increasing yields of mature granulocytes, the proportion of cells bearing Fc and C3 receptors increased. The most important finding was that the high- and low-receptor categories of CGL cells in chronic phase depended on the percentage of FcR+ cells. In the high-receptor CGL group, in addition to FcR, the proportion of CR1+ and CR2+ cells was also greater than in the low-receptor CGL group. Some differences in clinical course of both immunological CGL groups were observed.


*Br. J. Cancer* (1982) **45**, 194

### HIGH AND LOW Fc IgG-RECEPTOR EXPRESSION IN HUMAN CHRONIC GRANULOCYTIC LEUKAEMIA CELLS

A. HARŁOZIŃSKA*, J. POTOMSKI*, B. ŁAWINSKA†, A. NOWOROLSKA* AND R. RICHTER*

*From the *Department of Pathological Anatomy and the †Haematological Clinic, School of Medicine, 50–368 Wroclaw, Poland*

Received 22 January 1981 Accepted 29 September 1981

**Summary.—Discontinuous density-gradient centrifugation was used to separate chronic granulocytic leukaemia (CGL) cells in the chronic phase and blast crisis (BC) into fractions containing granulocytes in individual stages of maturation. The occurrence of the Fc IgG (FcR) and complement-component receptors (CR1 and CR2) in each fraction was estimated. It was established that, with increasing yields of mature granulocytes, the proportion of cells bearing Fc and C3 receptors increased. The most important finding was that the high- and low-receptor categories of CGL cells in chronic phase depended on the percentage of $FcR^+$ cells. In the high-receptor CGL group, in addition to FcR, the proportion of $CR1^+$ and $CR2^+$ cells was also greater than in the low-receptor CGL group. Some differences in clinical course of both immunological CGL groups were observed.**

LITERATURE about cell-membrane immunological markers during granulocyte differentiation, especially in different pathological states, is scarce. Since the acquisition of surface receptors is associated with immunological and functional differentiation, the identification of these structures may be helpful in studies of granulocyte physiology and classification of certain disease states (Ross *et al.*, 1978). In a previous study we have observed that AML blasts are poor in receptors (Harłozińska *et al.*, 1980). The number of cell-surface markers increases with differentiation (Rabellino *et al.*, 1978; Ross *et al.*, 1978; Berridge, 1979; Harłozińska *et al.*, 1980).

In the present study we have observed this process in CGL cells separated by discontinuous density-gradient centrifugation into fractions containing granulocytes in individual maturation stages, and have estimated the incidence of the Fc IgG (FcR) and complement-component C4b to C3b (CR1) and C3d (CR2) receptors. Emphasis was placed on determining the interrelationship of FcR expression, leukaemic granulocyte maturation and the clinical course of CGL.

### MATERIALS AND METHODS

*Human subjects.*—All immunological tests were performed on peripheral blood cells of 20 patients with CGL. Fourteen of these subjects were in the chronic phase of the disease, whilst 6 were in myeloblastic crises and made up a separate group. The diagnosis was established by standard morphological and cytochemical criteria. The cytochemical routine determinations included PAS reaction, lipid staining, acid and alkaline phosphatases activity, α-naphthyl acetate esterase and peroxidase.

In 11 patients with CGL in the chronic phase, immunological tests were performed directly after the diagnosis was established and before initiating treatment, whilst in 3 patients the tests were performed 2, 11 and 13 years after disease onset respectively.

Correspondence to: Dr A. Harłozińska, Department of Pathological Anatomy, School of Medicine, ul.Marcinkowskiego 1, 50–368 Wrocław, Poland.

The latter were under periodic clinical control and occasionally received small doses of busulphan. Of 6 patients with CGL-BC, 4 had the surface markers determined before treatment had begun, and in 2 this was performed 18 and 10 months after CGL diagnosis, when symptoms of blast transformation were observed. In the first of these patients polycytostatic treatment was started 1 week before the immunological tests were initiated, whilst the second patient received only small doses of busulphan at the moment of marker estimation.

The control studies were performed on blood cells from normal healthy volunteers 20–45 years old.

*Separation of cells.*—The leukaemic and normal blood cells were isolated from fresh, heparinized blood, mixed with an equal volume of 3% dextran T 500 (Pharmacia, Sweden) and left standing for 30 min at room temperature for sedimentation of erythrocytes. The leucocyte suspension were then separated into fractions by discontinuous density-gradient centrifugation according to Ross *et al.* (1978), slightly modified (Harłozińska *et al.*, 1980). Ficoll–Uropoline gradients, containing 7 density steps over the range 1·05–1·12, were used to separate myeloid cells according to the maturation stage associated with different densities. After centrifugation at 1200 *g* for 45 min, the cell layers concentrated at each Ficoll–Uropoline density interface were aspirated, washed with PBS, and counted. Viability of cells was determined after staining the preparations with 0·1% trypan blue. The cells of each density layer were stained with Wright–Giemsa to determine differential morphology.

*Rosette-forming cells.*—Cells with Fc receptors for IgG were detected by their capacity to form rosettes with sheep erythrocytes coated with 7S IgG anti-SRBC antibody (EA rosettes) according to Stingl & Knapp (1977) as earlier described in detail (Harłozińska *et al.*, 1980). Cells with C3 receptors were detected by their capacity to form rosettes with SRBC coated with 19S anti-SRBC antibody and complement (EAC rosettes) (Gupta *et al.*, 1976). Fresh serum of young BALB/c mice was used as a source of C3d ($EAC_{mo}$—CR2) and fresh rabbit serum as a source of C3b ($EAC_{ra}$—CR1) (Whiteside *et al.*, 1977). The methods of detecting both types of EAC rosettes were described in a previous study (Harłozińska *et al.*, 1980). In some cases differential counts of rosetted cells in fractions of individual density layers were made from Wright–Giemsa-stained smears. For staining, rosette mixtures were centrifuged at 100 *g* for 3 min, resuspended in 20 μl of foetal calf serum, smeared on glass slides, dried and then stained with Wright–Giemsa (Ross *et al.*, 1978).

### RESULTS

Ficoll–Uropoline gradient permitted the separation of the myeloid cell series according to the degree of morphological maturation, so that young forms of granulocytes were concentrated in low-density fractions, whereas polymorphonuclear neutrophils were restricted to high-density fractions (Tables I, II). This method yielded sufficient immature cells for surface-marker analysis. Low-density fractions contained 20–30% myeloblasts: fractions at density 1·06–1·07 focused most of the myelocytes and metamyelocytes, and fractions at density 1·08–1·105 concentrated band forms and polymorphs. The vast majority of lymphocytes and nucleated erythroid cells were isolated in the low-density layers (1·05–1·06) and were easily distinguished from large young forms of granulocytes by phase-contrast microscopy. Each fraction was defined morphologically by Wright–Giemsa staining. The expression of FcR and receptors for complement components (CR1 and CR2) were estimated in CGL and CGL-BC cells of individual maturation stages. On the basis of these studies it was possible to distinguish CGL patients (Table III) according to the number of cells expressing receptor for the Fc IgG fragment. A separate group of patients had the myeloblastic type of CGL-BC (Table III).

From the data in Table III it can be seen that the change in Fc IgG receptor values in cells of CGL patients does not depend on morphological differences of the leukaemic cell population, since the population after dextran isolation and the morphological composition of the

TABLE I.—*Detection of surface markers in high-receptor CGL cells separated by density-gradient centrifugation from the peripheral blood of Patient O.N.*

| Density layer (g/ml) | Number of cells ($\times 10^6$) | Wright–Giemsa morphology (%)* | | | | | | | Rosette-forming cells (%) | | |
|---|---|---|---|---|---|---|---|---|---|---|---|
| | | Blast | Pro | Myel | Mta | Band | PMN | Lym | EA | $EAC_{mo}$ | $EAC_{ra}$ |
| Dextran | 270·0 | 4 | 0 | 13 | 11 | 24 | 44 | 4 | 66·5 | 24·5 | 57·5 |
| 1·05 | 0·4 | 35 | 13 | 16 | 12 | 8 | 9 | 7 | 43·5 | ND | ND |
| 1·06 | 0·75 | 1 | 1 | 31 | 36 | 19 | 9 | 3 | 49·0 | ND | ND |
| 1·07 | 1·16 | 1 | 5 | 30 | 30 | 20 | 12 | 2 | 54·0 | 9·0 | ND |
| 1·08 | 14·0 | 0 | 0 | 2 | 12 | 45 | 41 | 0 | 65·0 | 23·0 | 53·5 |
| 1·09 | 28·0 | 1 | 0 | 0 | 7 | 31 | 60 | 1 | 69·5 | 44·0 | 75·0 |
| 1·105 | 15·6 | 1 | 0 | 0 | 2 | 25 | 72 | 0 | 85·0 | 29·0 | 86·5 |

* Blast = myeloblast; Pro = promyelocyte; Myel = myelocyte; Mta = metamyelocyte; Band = band form; PMN = polymorphonuclear neutrophil; Lym = lymphocyte; ND = not done (too few cells).

TABLE II.—*Detection of surface markers in low-receptor CGL cells separated by density-gradient centrifugation from the peripheral blood of Patient M.A.*

| Density layer (g/ml) | Number of cells ($\times 10^6$) | Wright–Giemsa morphology (%) | | | | | | | Rosette-forming cells (%) | | |
|---|---|---|---|---|---|---|---|---|---|---|---|
| | | Blasts | Pro | Myel | Mta | Band | PMN | Lym | EA | $EAC_{mo}$ | $EAC_{ra}$ |
| Dextran | 1370·0 | 2 | 1 | 27 | 13 | 28 | 24 | 5 | 7·0 | 3·0 | 8·5 |
| 1·05 | 24·0 | 35 | 5 | 51 | 2 | 3 | 2 | 2 | 1·0 | 0·0 | 0·0 |
| 1·06 | 9·2 | 20 | 6 | 30 | 10 | 10 | 18 | 6 | 3·0 | 2·0 | 0·0 |
| 1·07 | 15·4 | 0 | 0 | 59 | 20 | 10 | 8 | 2 | 3·0 | 2·0 | 2·0 |
| 1·08 | 69·6 | 0 | 1 | 2 | 5 | 28 | 63 | 63 | 13·5 | 3·5 | 7·0 |
| 1·09 | 66·0 | 0 | 0 | 2 | 4 | 24 | 70 | 0 | 16·0 | 4·0 | 12·0 |
| 1·105 | 26·4 | 0 | 0 | 1 | 5 | 26 | 68 | 0 | 16·0 | 4·0 | 17·0 |

individual fractions of discontinuous density gradient were basically similar in all 3 groups of patients. Fig. 1 shows the comparative values for FcR in individual patients in the high- and low-receptor CGL groups in the chronic phase of the disease, in the CGL-BC group and in normal leucocytes. The data represent results after dextran T 500 cell isolation. As can be seen, even without cell fractionation, certain differences are evident in the proportion of $FcR^+$ cells. The lowest marker content was found in the low-receptor CGL group, intermediate content in cells of CGL-BC group, and higher still in the high-receptor group of CGL cases. The highest FcR content is found in leucocytes from normal donors.

A similar distribution is obtained on estimating the C3 components. Fig. 2 illustrates the comparison of means $\pm$ s.d. for all studied markers in both groups of patients in the chronic phase of CGL, in those with CGL-BC and in normal donors. The lowest spread of values was in the low-receptor CGL, the highest in the high-receptor group and CGL-BC. As can be seen from Tables I, III and Fig. 2, CGL cases with a high proportion of FcR-bearing cells usually contain a high number of cells expressing both complement components, and the number of cells expressing all 3 markers distinctly increased in high-density fractions with more mature neutrophils. In this group of leukaemias the distribution of surface markers in individual density fractions was similar to that of normal leucocytes.

Table I shows a representative CGL case whose cells have a high proportion of surface markers. The earliest marker was FcR, whose presence could be demonstrated in low-density fractions containing mainly immature granulocytes (Fraction 1·05). Cytocentrifuge preparations after Wright–Giemsa staining of CGL cells indicated that on the average 14% of the myeloblasts expressed FcR. This con-

TABLE III.—*Comparative results of surface markers in leukaemic and normal cells separated by density-gradient centrifugation*

| Material | Number of cases | Density layer (g/ml) | Wright–Giemsa morphology (mean%) Blasts Pro | Myel Mta | Band PMN | Lym | Mono | Surface markers (mean%) FcR | CR2 | CR1 |
|---|---|---|---|---|---|---|---|---|---|---|
| 1. High-receptor CGL cells (FcR > 25%) | 8 | Dextran | 5·2 | 31·0 | 61·9 | 1·9 | 0 | 44·7 | 24·9 | 31·4 |
| | | 1·05 | 30·1 | 53·9 | 11·1 | 4·9 | 0 | 19·6 | 5·5 | 4·8 |
| | | 1·06–1·07 | 5·2 | 70·9 | 22·4 | 1·5 | 0 | 31·0 | 14·4 | 15·2 |
| | | 1·08–1·09 | 0·6 | 14·8 | 84·0 | 0·6 | 0 | 40·1 | 21·3 | 27·4 |
| | | 1·105 | 0·4 | 8·2 | 91·0 | 0·4 | 0 | 59·6 | 24·4 | 52·3 |
| 2. Low-receptor CGL cells (FcR < 20%) | 6 | Dextran | 10·0 | 22·0 | 63·5 | 4·5 | 0 | 7·5 | 5·0 | 5·4 |
| | | 1·05 | 16·3 | 51·0 | 31·0 | 1·7 | 0 | 2·2 | 1·7 | 0·5 |
| | | 1·06–1·07 | 5·9 | 68·9 | 24·1 | 1·1 | 0 | 4·8 | 2·9 | 1·2 |
| | | 1·08–1·09 | 0·9 | 24·4 | 73·2 | 1·5 | 0 | 10·8 | 5·0 | 5·4 |
| | | 1·105 | 4·0 | 10·4 | 85·6 | 0·2 | 0 | 15·9 | 7·9 | 12·3 |
| 3. CGL in blast crisis | 6 | Dextran | 24·0 | 17·0 | 52·0 | 7·0 | 0 | 12·8 | 7·8 | 14·8 |
| | | 1·05 | 40·3 | 52·0 | 7·4 | 0·3 | 0 | 8·6 | 3·7 | 4·0 |
| | | 1·06–1·07 | 26·5 | 56·0 | 17·0 | 0·5 | 0 | 9·3 | 4·7 | 5·8 |
| | | 1·08–1·09 | 4·2 | 31·5 | 63·5 | 0·8 | 0 | 15·4 | 9·5 | 18·5 |
| | | 1·105 | 0·0 | 3·0 | 97·0 | 0 | 0 | 19·0 | 10·0 | 18·0 |
| 4. Normal leucocytes | 10 | Dextran | 0 | 2·0 | 70·0 | 27·0 | 1·0 | 64·6 | 20·9 | 59·7 |
| | | 1·05 | 1·5 | 0·5 | 3·0 | 95·0 | 0 | ND | ND | ND |
| | | 1·06–1·07 | 0·5 | 1·0 | 20·0 | 78·5 | 0 | ND | ND | ND |
| | | 1·08 | 0 | 0·3 | 50·6 | 49·1 | 0 | 45·3 | 21·8 | 34·5 |
| | | 1·09 | 0 | 1·0 | 86·8 | 11·2 | 1·0 | 64·9 | 37·9 | 57·4 |
| | | 1·105 | 0 | 0·5 | 95·0 | 3·5 | 1·0 | 72·6 | 19·1 | 67·6 |

firmed the very early appearance of that marker in the maturation process of the myeloid cell line. The number of cells expressing that marker distinctly increased as the mature granulocyte fractions were obtained. Receptors for both complement components were present on a low percentage of young forms of its myeloid cell series (Table III) and the number of $CR1^+$ cells increased more distinctly than $CR2^+$ as more neutrophils matured.

The second group of leukaemias, designated as low-receptor CGL, was characterized by a low proportion of FcR ( < 20% in the leucocyte fraction after dextran isolation). The cell number expressing this receptor steadily increased as more mature cells in denser layers were obtained, but the percentage was always much lower than in the high-receptor leukaemias (Table III). Similarly, only a small proportion of cells expressed C3 receptors. The distribution of the surface markers in this CGL group was similar to the marker values in patients in blast crisis.

Our observations indicate that high-receptor CGL may have a slightly better clinical prognosis. Up to the present, only 2 patients from the high-receptor group have died. In the first patient symptoms of an unresponsive blast crisis appeared 19 months after diagnosis, whilst the second patient died without signs of exacerbation after 13·5 years. The rest of the patients in this group have lived for 11, 3, 2·5 and 2 years and 3 and 2 months respectively. Only in one of these patients (on polycytostatic therapy) was there a blast crisis after 2 years of disease. At the time of writing the 6 surviving patients are in a good clinical and haematological state.

In the low-marker group 2 patients died 13 and 18 months after diagnosis, due to a treatment-resistant blast crisis. A third patient showed signs of exacerbation 11 months after diagnosis of CGL, and is in a grave general state. One female patient from this group is alive after 2·5 years and her condition is satisfactory. In 2 remaining patients the marker analysis was performed 2 months after the diagnosis.

FIG. 1.—Distribution of FcR in leucocytes of individual patients with low- and high-receptor CGL in chronic phase, CGL-BC, and normal leucocytes (dextran isolation).

In the CGL-BC group, 4 patients have died so far at 4, 6, 23 and 26 months from diagnosis. In the 2 remaining the blast crisis was diagnosed and immunological determinations were performed only in the last 3 months.

### DISCUSSION

According to our immunological findings we have been able to distinguish 2 subgroups of CGL: one with high-receptor cells and the other with low-receptor cells.

The most characteristic marker for such division was the Fc receptor for IgG.

In high-receptor leukaemias, an average of 20% of immature cells, isolated in low-density layers, expressed this receptor, and that percentage increased gradually to about 60% in the high-density layers containing morphologically mature polymorphs.

In low-receptor leukaemias, the FcR was detectable on ~2% of immature granulocytes obtained in low-density layers, and at higher densities the percentage of $FcR^+$ cells increased on the average to only 16% (Table III).

The possible phenotypic heterogeneity of CGL is already seen in the FcR determinations in cells isolated on dextran T 500, which is independent of granulocyte separation according to morphological maturity.

Similar, but less distinct, differences were found on estimating the proportion of cells expressing receptors for complement components. In high-receptor CGL leukaemias the proportion of cells with CR1 and CR2 receptors was always much higher than in low-receptor CGL.

On the basis of observations up to the present time, it seems that high-receptor

FIG. 2.—Fc (■) and C3 (CR2, ▲; CR1, ●) receptors in low- and high-receptor CGL cells in chronic phase, CGL-BC cells and normal leucocytes. Vertical bars indicate s.d. around means.

CGL patients, containing in their peripheral blood immunologically mature neutrophils, with a proportion of surface markers similar to normal granulocytes, had a better clinical prognosis, and that blast crisis or concomitant infections were rare. This allows us to assume that the dominance of immunologically immature malignant clones or subclones in the picture of peripheral blood is connected with a more acute and heavy course of CGL.

Recently Palu *et al.* (1979*a,b*) have shown that the ability of $Fc^-$ AML blasts to induce neoplasms in mice with a weakened immunological system distinctly decreases with the acquisition of Fc and C3 receptors on the surface of cells cultured *in vitro*.

A fundamental question raised by our studies is the origin of high- and low-receptor CGL cells. Several possibilities may be considered, including the existence of subpopulations with different frequencies of EA and EAC rosettes or blocks that may occur on different maturation stages of myeloid cells. Some data indicate that human neutrophils comprise heterogeneous populations (Klempner & Gallin, 1978) and Sachs (1977) has developed an experimental system for cloning myeloid leukaemia cells with different capacities for EA- and/or EAC-rosette formation.

The density-gradient technique appeared to be valuable for concentrating immature granulocytes (Ross *et al.*, 1978). Estimation of Fc receptor in these fractions confirmed its presence at an early phase of neutrophil differentiation (Herborn *et al.*, 1979; Harłozińska *et al.*, 1980).

$Fc^+$ cells increased steadily, reaching the highest proportion in fractions with mature polymorphs.

In a previous paper (Harłozińska *et al.*, 1980) we showed that the appearance of CR1 was preceded by a CR2-type receptor. Now we have demonstrated that both types of complement receptors, in all tested groups, increased up to the fraction of density 1·09, and in fraction 1·105 contained the denser polymorphs, whilst the number of $CR2^+$ cells does not change significantly, but the amount of $CR1^+$ cells continued to rise. In normal leucocytes and in some cases of "high-receptor" myeloid leukaemias, the amount of $CR2^+$ cells in the 1·105 fraction was significantly reduced. These observations are consistent with the data of Ross *et al.* (1978) indicating that less dense neutrophils, containing both CR1 and CR2 receptors, differed in the mechanism of EAC-rosette formation from their denser counterparts.

Our studies—especially the demonstration of high- and low-receptor CGL forms, despite the similar morphological patterns in individual density layers—indicate that regulation of membrane immunological differentiation may be independent from maturation as defined by standard morphological criteria.

This work was supported by the Polish National Cancer Programme PR-6, Grant No. 1304/13.

### REFERENCES

Berridge, M. V. (1979) A new class of cell surface antigens. Quantitative absorption studies defining cell-lineage-specific antigens on haemopoietic cells. *J. Exp. Med.*, **150**, 977.

Gupta, S., Good, R. A. & Siegal, F. P. (1976) Rosette-formation with mouse erythrocytes. II. A marker for human B and non-T-lymphocytes. *Clin. Exp. Immunol.*, **25**, 319.

Harłozińska, A., Potomski, J., Noworolska, A., Becker, M. & Richter, R. (1980) Fc and C3 receptors as membrane differentiation markers of acute myelogenous leukaemia cells. *Scand. J. Haematol.*, **25**, 205.

Herborn, H. A., Valdimarsson, H. & Wickramasinghe, S. N. (1979) Development of human granulocyte and monocyte Fc receptors. *Scand. J. Haematol.*, **22**, 364.

Klempner, M. S. & Gallin, J. I. (1978) Separation and functional characterization of human neutrophil subpopulations. *Blood*, **51**, 659.

Palu, G., Powles, R., Selby, P., Summersgill, B. M. & Alexander, P. (1979*a*) Patterns of maturation in short-term culture of human acute myeloid leukaemic cells. *Br. J. Cancer*, **40**, 719.

Palu, G., Selby, P., Powles, R. & Alexander, P. (1979*b*) Spontaneous regression of human acute myeloid leukaemia xenografts and phenotypic evidence for maturation. *Br. J. Cancer*, **40**, 731.

Rabellino, E. M., Ross, G. D., Trang, H. T. K., Williams, N. & Metcalf, D. (1978) Membrane receptors of mouse leukocytes. III. Sequential expression of membrane receptors and phagocytic capacity during leukocyte differentiation. *J. Exp. Med.*, **147**, 434.

Ross, G. D., Jarowski, C. I., Rabellino, E. M. & Winchester, R. J. (1978) The sequential appearance of Ia-like antigens and two different complement receptors during the maturation of human neutrophils. *J. Exp. Med.*, **147**, 730.

Sachs, L. (1977) Control of normal cell differentiation in leukemia. *Isr. J. Med. Sci.*, **13**, 654.

Stingl, G. & Knapp, W. (1977) Characterization of neoplastic mononuclear cells on tissue sections. In *Immunological Diagnosis of Leukemias and Lymphomas, Vol. 20* (Eds. Thierfelder *et al.*). Berlin: Springer Verlag. p. 189.

Whiteside, T. L., Winkelstein, A. & Rabin, B. S. (1977) Immunological characterization of chronic lymphocytic leukemia cells. *Cancer*, **39**, 1109.


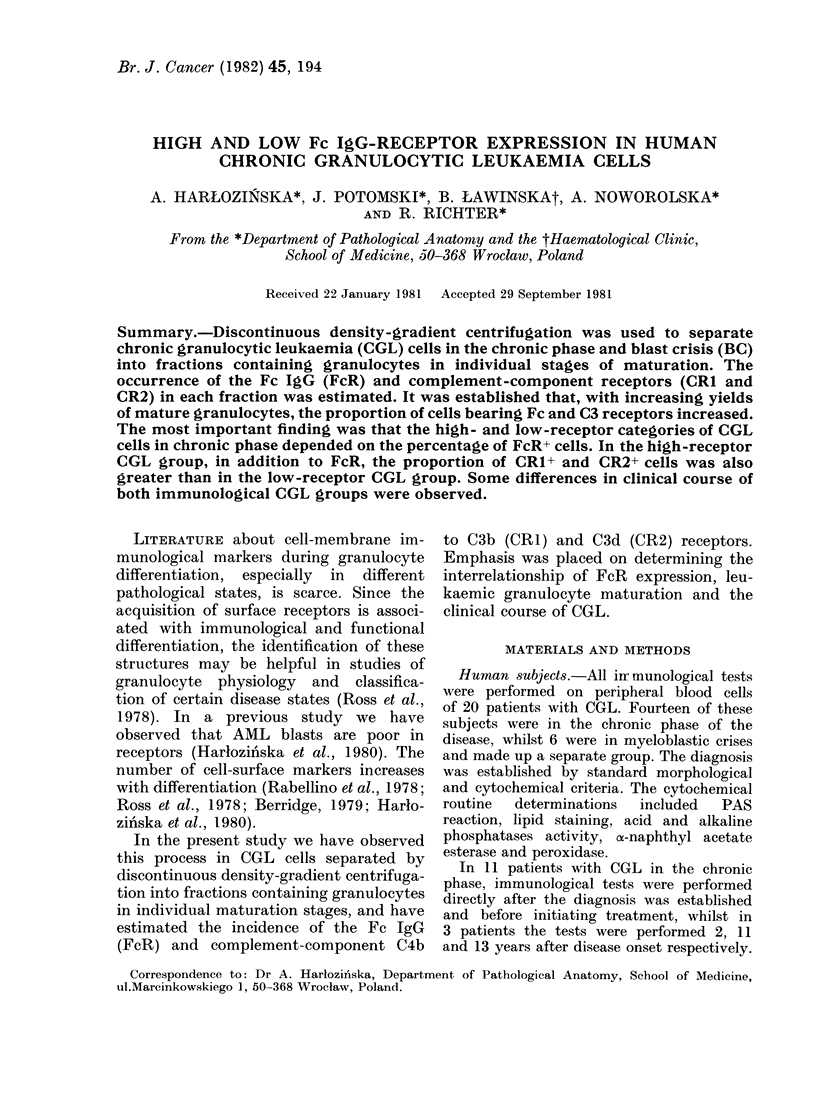

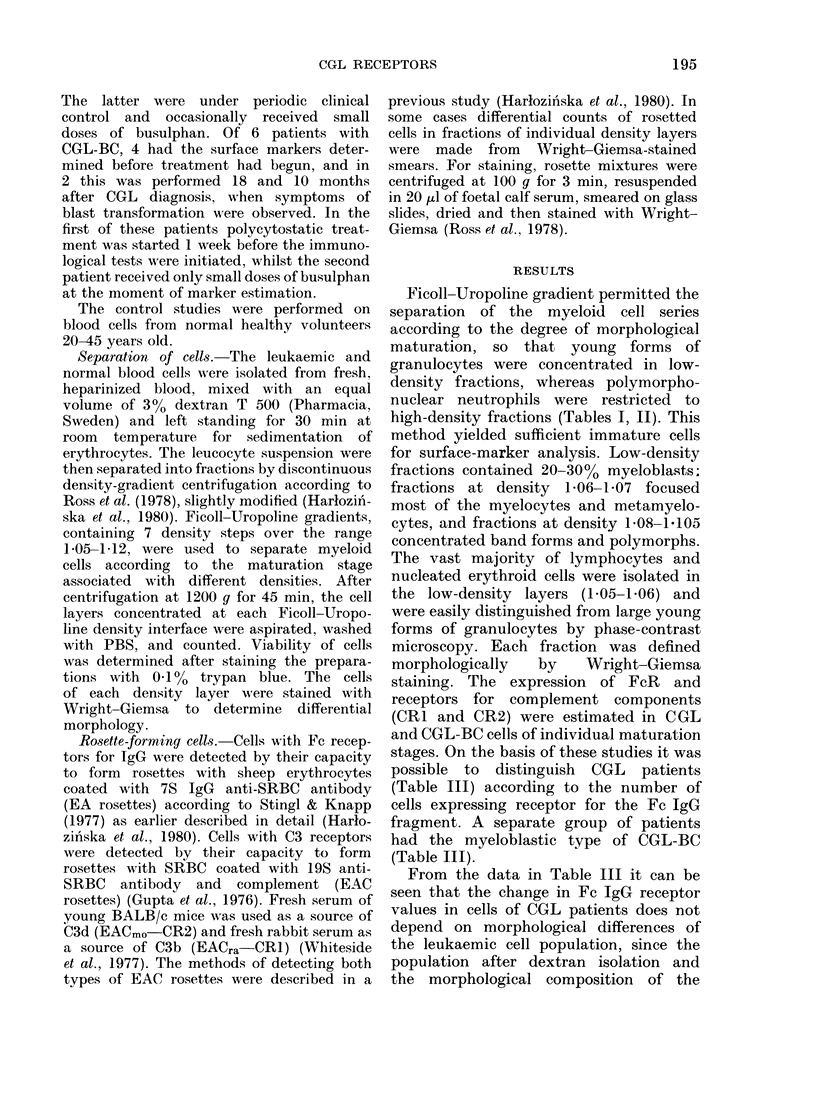

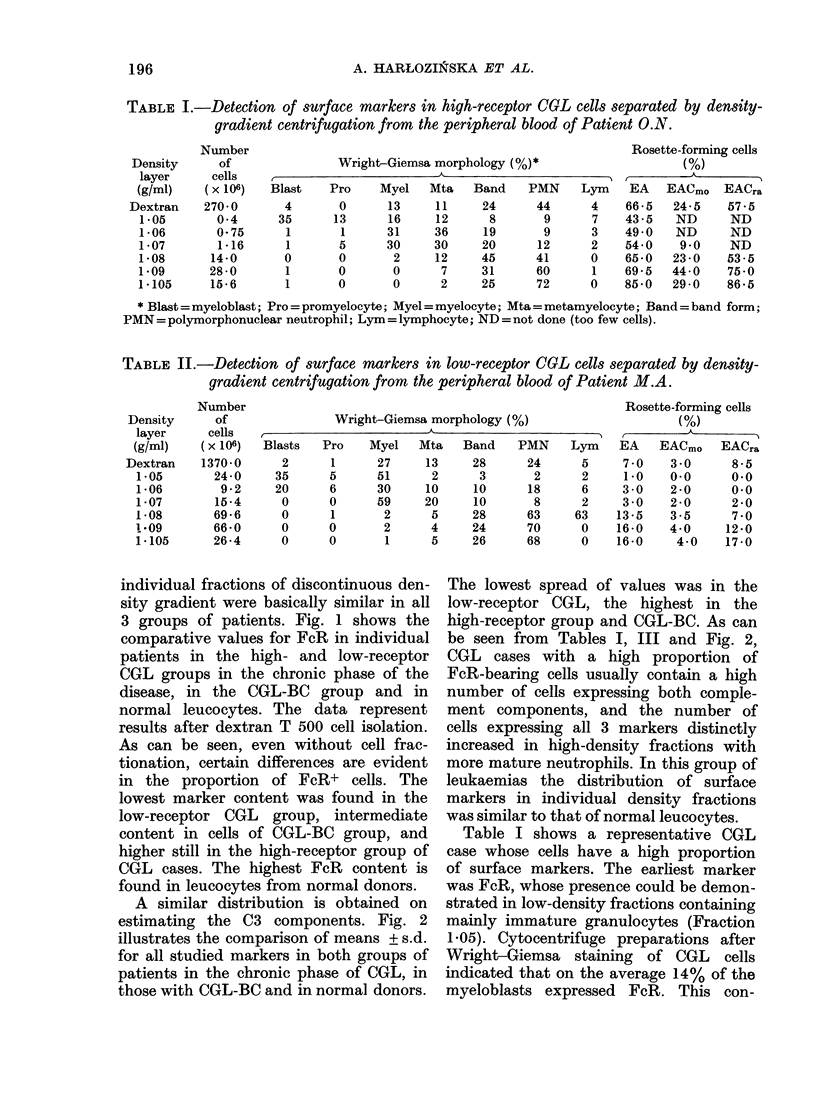

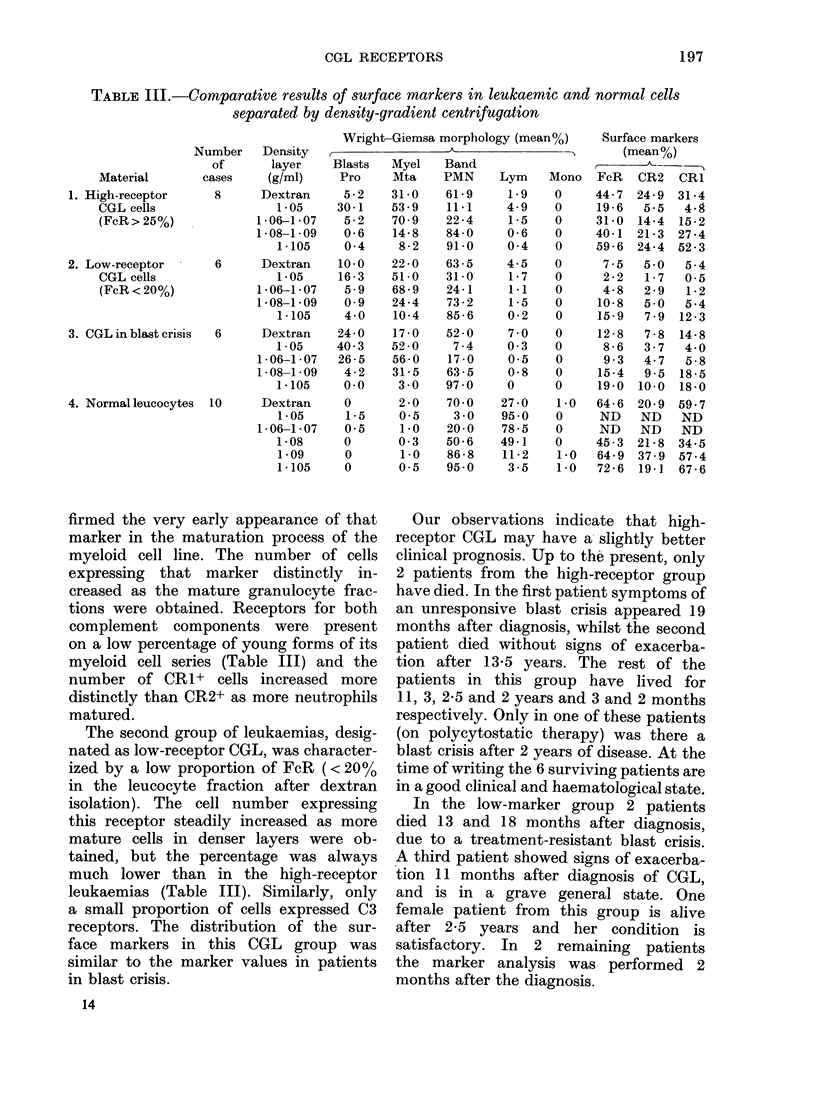

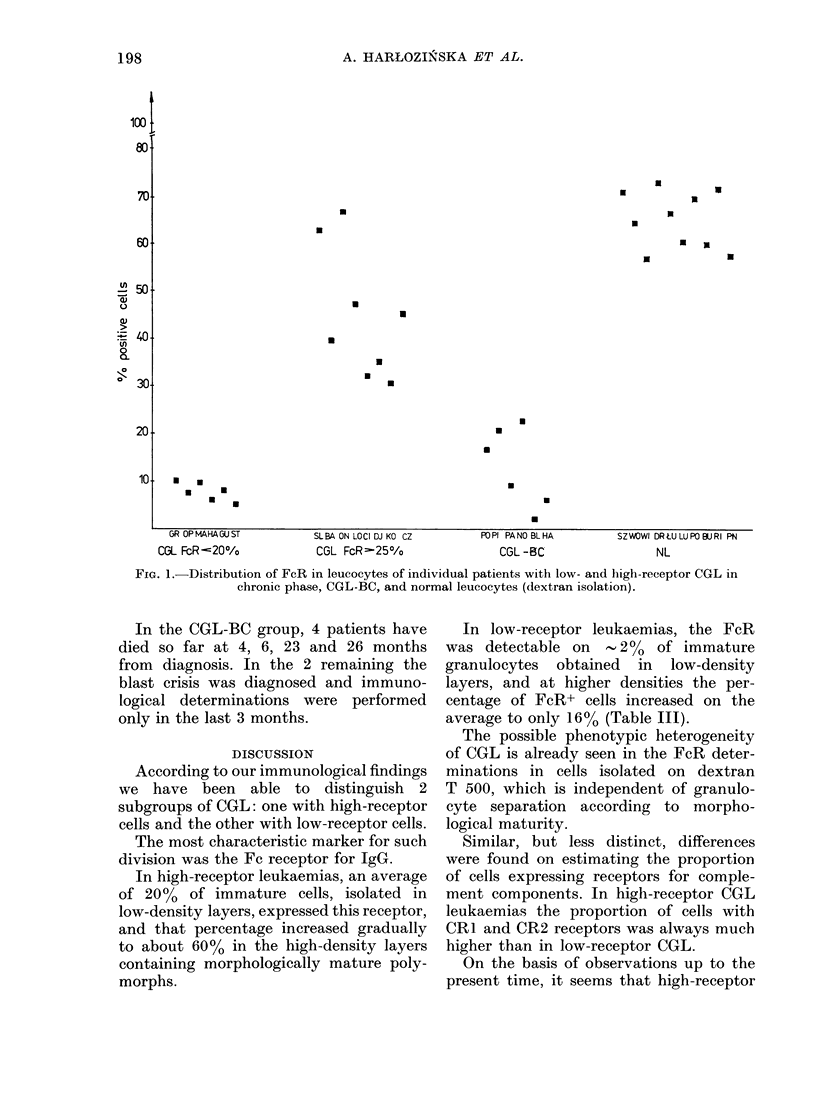

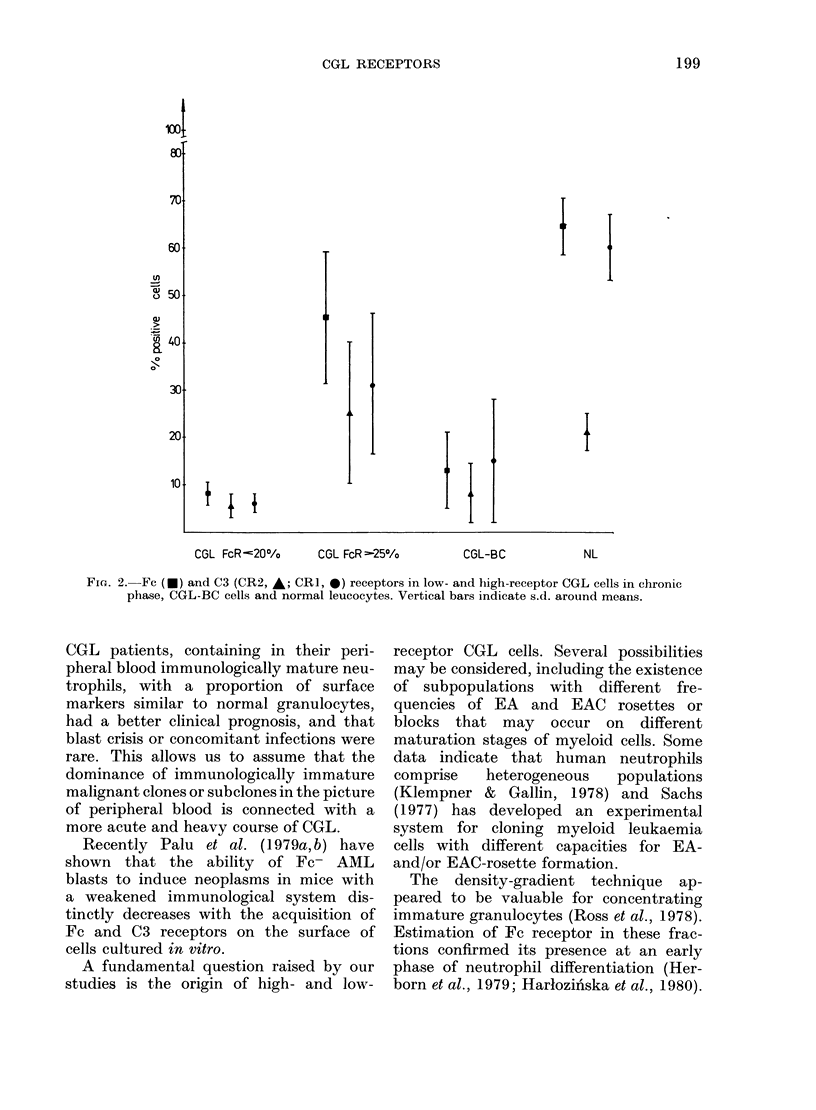

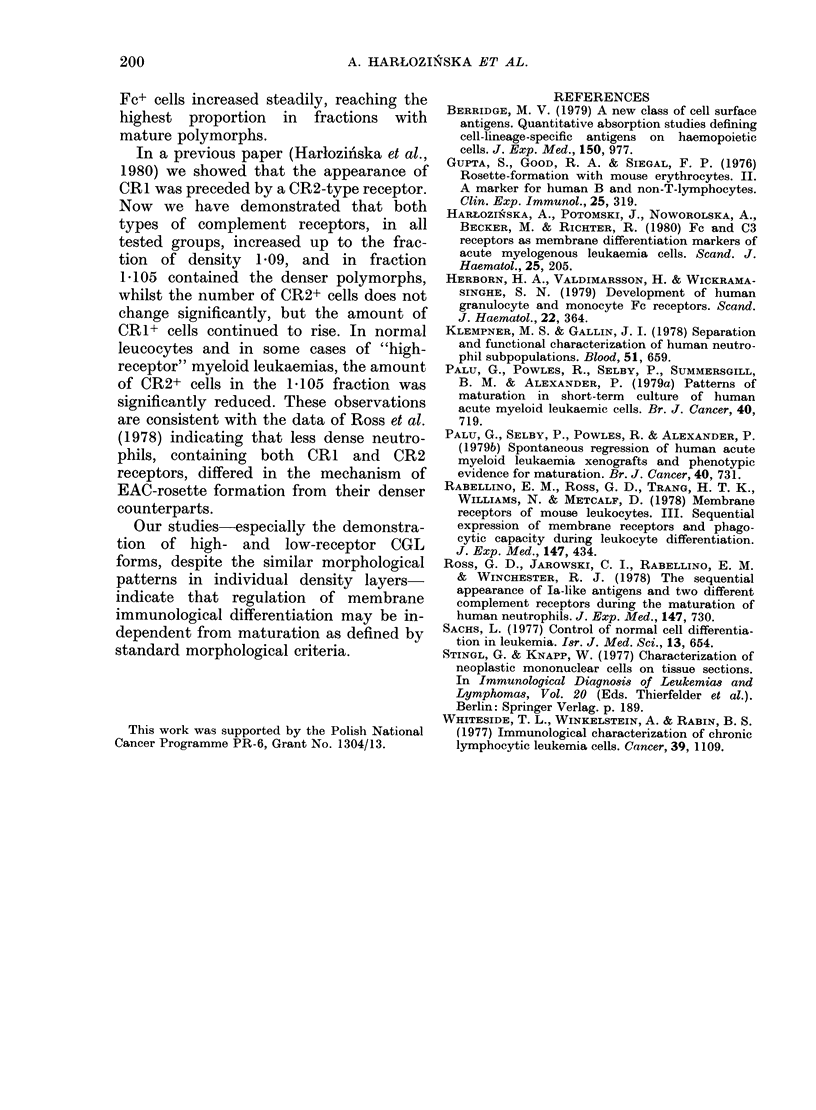


## References

[OCR_01018] Berridge M. V. (1979). A new class of cell surface antigens. Quantitative absorption studies defining cell-lineage-specific antigens on hemopoietic cells.. J Exp Med.

[OCR_01024] Gupta S., Good R. A., Siegal F. P. (1976). Rosette-formation with mouse erythrocytes. II. A marker for human B and non-T lymphocytes.. Clin Exp Immunol.

[OCR_01030] Harlozinska A., Potomski J., Noworolska A., Becker M., Richter R. (1980). Fc and C3 receptors as membrane differentiation markers of acute myelogenous leukaemia cells.. Scand J Haematol.

[OCR_01039] Herborn H. A., Valdimarsson H., Wickramasinghe S. N. (1979). Development of human granulocyte and monocyte Fc receptors.. Scand J Haematol.

[OCR_01043] Klempner M. S., Gallin J. I. (1978). Separation and functional characterization of human neutrophil subpopulations.. Blood.

[OCR_01048] Palú G., Powles R., Selby P., Summersgill B. M., Alexander P. (1979). Patterns of maturation in short-term culture of human acute myeloid leukaemic cells.. Br J Cancer.

[OCR_01061] Rabellino E. M., Ross G. D., Trang H. T., Williams N., Metcalf D. (1978). Membrane receptors of mouse leukocytes. II. Sequential expression of membrane receptors and phagocytic capacity during leukocyte differentiation.. J Exp Med.

[OCR_01069] Ross G. D., Jarowski C. I., Rabellino E. M., Winchester R. J. (1978). The sequential appearance of Ia-like antigens and two different complement receptors during the maturation of human neutrophils.. J Exp Med.

[OCR_01087] Whiteside T. L., Winkelstein A., Rabin B. S. (1977). Immunologic characterization of chronic lymphocytic leukemia cells.. Cancer.

